# CD117 Expression in Fibroblasts-Like Stromal Cells Indicates Unfavorable Clinical Outcomes in Ovarian Carcinoma Patients

**DOI:** 10.1371/journal.pone.0112209

**Published:** 2014-11-07

**Authors:** Ruixia Huang, Dan Wu, Yuan Yuan, Xiaoran Li, Ruth Holm, Claes G. Trope, Jahn M. Nesland, Zhenhe Suo

**Affiliations:** 1 Departments of Pathology, The Norwegian Radium Hospital, Oslo University Hospital, Oslo, Norway; 2 Department of Pathology, Institute of Clinical Medicine, Faculty of Medicine, University of Oslo, Oslo, Norway; 3 Department of Gynecology, International Peace Maternity and Child Health Hospital, Medical College of Shanghai, Jiaotong University, Shanghai, China; 4 Department of Pathology, Capital Medical University, Beijing, China; 5 Department of Gynecology, Institute of Clinical Medicine, Faculty of Medicine, University of Oslo, Oslo, Norway; 6 Department of Gynecology, The Norwegian Radium Hospital, Oslo University Hospital, Oslo, Norway; Seoul National University, Republic Of Korea

## Abstract

The stem cell factor (SCF) receptor CD117 (c-kit), is widely used for identification of hematopoietic stem cells and cancer stem cells. Moreover, CD117 expression in carcinoma cells indicates a poor prognosis in a variety of cancers. However the potential expression in tumor microenvironment and the biological and clinical impact are currently not reported. The expression of CD117 was immunohistochemically evaluated in a serial of 242 epithelial ovarian cancer (EOC) cases. Thirty-eight out of 242 cases were CD117 positive in fibroblast-like stromal cells and 22 cases were positive in EOC cells. Four cases were both positive in fibroblast-like stromal cells and EOC cells for CD117. CD117 expression in fibroblast-like stromal cells in ovarian carcinoma was closely linked to advanced FIGO stage, poor differentiation grade and histological subtype (*p*<0.05), and it was significantly associated with poor overall survival (OS) and progression free survival (PFS) (Kaplan-Meier analysis; *p*<0.05, log-rank test). CD117 expression in ovarian carcinoma cells was not associated with these clinicopathological variables. The CD117 positive fibroblast-like stromal cells were all positive for mesenchymal stem/stromal cell (MSC) marker CD73 but negative for fibroblast markers fibroblast activation protein (FAP) and α smooth muscle actin (α-SMA), indicating that the CD117+/CD73+ fibroblast-like stromal cells are a subtype of mesenchymal stem cells in tumor stroma, although further characterization of these cells are needed. It is concluded herewith that the presence of CD117+/CD73+ fibroblast-like stromal cells in ovarian carcinoma is an unfavorable clinical outcome indication.

## Introduction

Epithelial ovarian cancer (EOC) is the most lethal gynecologic malignancy worldwide [1], [2]. Most women with EOC are diagnosed at an advanced stage. The 5-year survival is less than 30% for the women diagnosed at advanced stage [2]. Cancer stem cells (CSCs) represent a subpopulation of tumor cells responsible for tumor initiation, progression, invasion, metastasis and relapse, and are capable of cell renewal. Thus, therapies targeting CSCs are being considered a promising way to control cancer.

Tumor microenvironment is the cellular environment in which tumor cells exist, including blood vessels, immune cells, fibroblasts, macrophages, extracellular matrix (ECM) and other molecules, etc. The stromal cells interact with each other in a complex net by secreting distinct, abnormal molecules into ECM and contribute to tumor growth and invasiveness [3],[4],[5],[6],[7]. Tumor stromal cells from different tissue have different gene expression profiles and hence contribute to tumor heterogeneity [8]. According to Albini, et. al, the microenvironment of a cancer is an integral part of its anatomy and physiology, and functionally, one cannot totally dissociate this microenvironment from what have traditionally been called “cancer cells” [9]. Therefore, it is now necessary to develop new tumorpreventive methods to target tumor microenvironment and hence to interfere carcinogenesis [9].

CD117, also known as proto-oncogene c-Kit or tyrosine-protein kinase Kit, is a transmembrane cytokine receptor expressed on the surface of hematopoietic stem cells and other cell types. It is normally phosphorylated and activated by binding to its ligand stem cell factor (SCF). It is a widely used stemness marker for recognition of cancer stem cells in various tumors, including ovarian carcinoma, endometrial cancer, osteosarcoma [10], [11], [12] and others. The unfavorable outcome for cancer patients with CD117 expression in cancer cells is well established [10], [11], whereas, the role of CD117 expression in stromal cells is rarely reported.

To better understand CD117 expression in ovarian carcinoma cells and stromal cells and the clinicopathological associations, we investigated CD117 expression in 242 formalin-fixed paraffin-embedded (FFPE) tumor tissue samples, which were obtained routinely from surgical dissection of patients diagnosed with EOC in The Norwegian Radium Hospital, Oslo University Hospital. Furthermore, we analyzed the associations between CD117 expression and clinicopathological characters, including age, histological subtype, differentiation grade, FIGO stage, overall survival (OS) and progression free survival (PFS). CD117 expressions in stromal cells and tumor cells and the clinicopathological analyses were investigated respectively.

## Materials and Methods

### Ethics Statement

The Regional Committee for Medical Research Ethics South of Norway (S-06277a), The Social- and Health Directorate (06/3280) and The Data Inspectorate (06/5345) approved the study. All the patients involved provided their written consent to participate in this study, and all the written consents were filed in The Norwegian Radium Hospital, Oslo University Hospital.

### Clinical samples

Two-hundred and forty-two surgically dissected ovarian carcinoma samples were randomly enrolled in this study. All patients were operated at The Norwegian Radium Hospital, Oslo University Hospital from March 1983 to May 2001. FFPE ovarian carcinoma tissues were obtained from the files of the Department of Pathology, and 3µm sections were cut and used for morphological examination and immunohistochemistry (IHC). The ages of the patients range from 19 to 89 years, and the median age is at 58 years old. The patients were followed up until January 1^st^ 2012. All patients were clinically staged following the recommendations of International Federation of Gynecology and Obstetrics (FIGO) [13]. The primary tumors were histologically graded as well, moderately and poorly differentiated according to WHO recommendations [13]. Disease progression was determined based on the definitions outlined by the Gynecologic Cancer Intergroup [14].

### Immunohistochemistry (IHC)

The Dako Envision FLEX+ system (K8012; Dako, Glostrup, Denmark) and the Dako Autostainer were used for IHC. Paraffin sections were deparaffinized and epitopes unmasked in PT link with high pH/low pH target retrieval solution (Dako), and then blocked with peroxidase blocking (Dako) for 5 minutes. The slides were incubated with primary antibody 30 minutes at room temperature, following up with rabbit/mouse linker (Dako) according to the resource of primary antibody for 15 minutes and HRP for 30 minutes at room temperature. Slides were then stained with 3, 3′-diaminobenzidine tetrahydrochloride (DAB) for 10 minutes and counter-stained with hematoxylin, dehydrated, and mounted in Richard-Allan Scientific Cyto seal XYL (Thermo Scientific, Waltham, MA, USA). Already known each antibody positive tissue was used as positive control in the same procedure. The same positive control slide was used as a negative control incubated with the same concentration of non-immune rabbit/mouse IgG replacing the primary antibody. The information for each primary antibody is shown in Table 1.

**Table 1:** The primary antibodies used for IHC.

Name	Company	Catalog number	Resource	Dilution	Retrieval solution	Positive control
CD117	Dako	A4502	Rabbit	1∶400	HPH	Human seminoma tissue
FAP	Abcam	Ab53066	Rabbit	1∶300	HPH	Human colon tissue
α-SMA	BioGenex	MU128-UC	Mouse	1∶750	HPH	Human appendix tissue
CD73	LSBio	LS-C138754	Rabbit	1∶1600	LPH	Human tonsil tissue

### IHC scoring system

CD117 immunodetection was evaluated and grouped as positive and negative in carcinoma cells and stromal cells for each slide. Slides were grouped as positive for tumor cells if over 10 percent of tumor cells were stained. Likewise, slides were grouped as positive for stromal cells if over 10 percent of stromal cells were stained. The morphology and immunostaining judgment were confirmed by two pathologists.

### Statistical analyses

SPSS software (version 18.0) was used for data analyses. Associations between categorical variables were assessed by Chi-square tests (Pearson and linear-by-linear as appropriate). Survival analysis was estimated using the Kaplan-Meier method, and groups were compared with log-rank tests. For all the analyses, associations were considered to be significant if the *p* value was <0.05.

## Results

### Immunodetection of CD117 in ovarian carcinoma tissues

**Figure 1:**
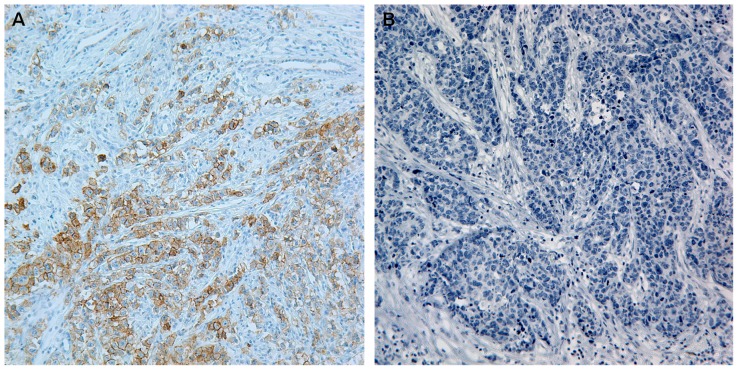
(A) Known CD117-positive seminoma tissue is always positive for CD117 and used as positive control in this study. (B) Section from the same seminoma tissue is negative by incubating with the corresponding non-immune rabbit IgG in the same concentration, instead of rabbit anti-CD117 antibody. Both pictures were taken at 200×.

**Figure 2:**
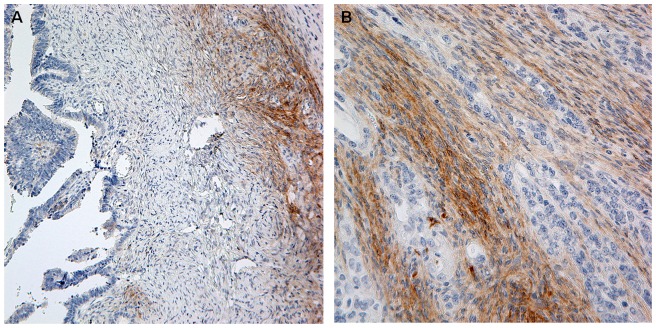
CD117 positive fibroblast-like stromal cells surrounding ovarian carcinoma cells were shown (A:200×, and B: 400×).

**Figure 3:**
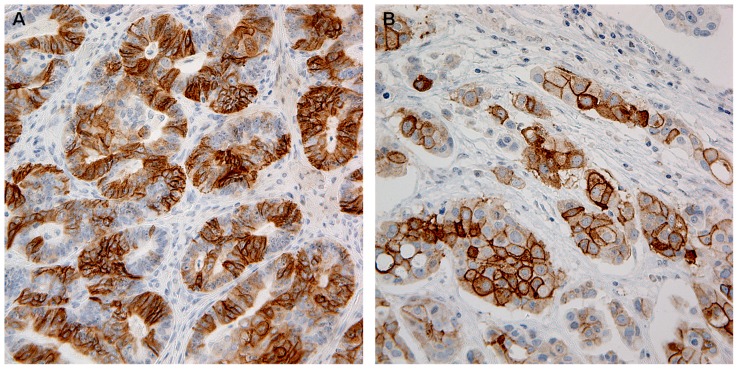
Variable expression of CD117 in ovarian carcinoma cells is demonstrated. The magnitude was 200× for (A) and 400× for (B).

**Figure 4:**
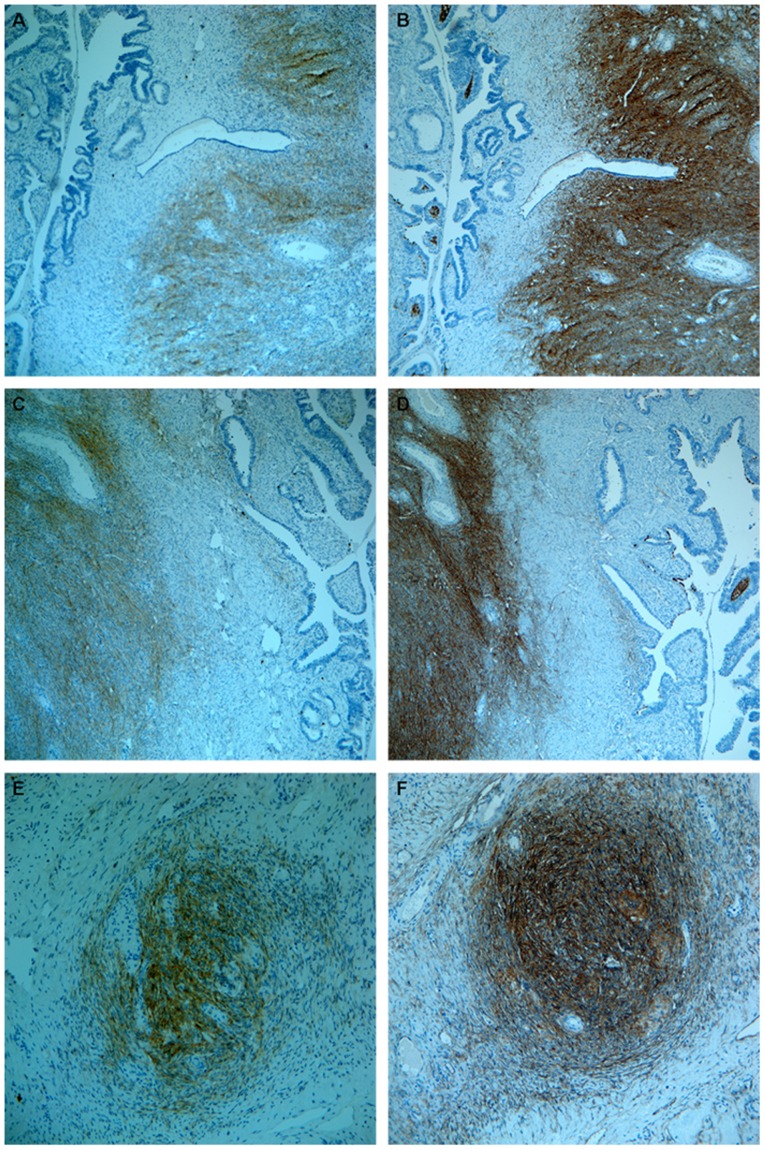
Three pairs of slides stained with CD117 and CD73 for each pair were randomly picked and demonstrated. Each pair of the slides was from the same tumor tissue in our database. CD117+ fibroblast-like stromal cells (A,C,E) were all positive for CD73 (B,D,F). (A,B,C,D) were taken at 100× and (E, F) were taken at 200×.

Generally, CD117 immunoreactivity was observed in some EOC cells and stromal cells, including endothelial cells, fibroblast-like stromal cells and blood cells. It was found out that out of 242 cases 38 were positive in fibroblast-like stromal cells and 22 cases were positive for CD117 in EOC cells. Four cases were positive in both fibroblast-like stromal cells and EOC cells. Positive control and negative control were shown in Figure 1. CD117 immnoreactivity was limited to cytoplasm and membrane in both fibroblast-like stromal cells (Figure 2) and tumor cells (Figure 3). Nuclear staining was not observed. It was commonly observed if possible, tumor cells in the same case were variably stained (Figure 3B), and some cases were scattered immunostaining. CD117 positive fibroblast-like stromal cells were further identified by mesenchymal stem/stromal cell (MSC) marker CD73 (NT5E) and cancer associated fibroblast (CAF) markers fibroblast activation protein (FAP) and α-smooth muscle actin (α-SMA). All CD117 positive stromal cells were negative for both FAP and α-SMA, but positive for CD73 (Figure 4).

### CD117 expression in fibroblast-like stromal cells and clinical impact

The associations between CD117 expression in fibroblast-like stromal cells and the clinicopathological variables are demonstrated in Table 2. CD117 expression in fibroblast-like stromal cells was significantly associated with late FIGO stage, poor differentiation stage, and histological subtypes (*p*<0.05). No significant difference was observed for age (patients diagnosed at ≥ 60 years and ≤59 years (*p* = 0.411)). CD117 expression in fibroblast-like stromal cells was observed in 4.3% (2/46) of EOC patients in FIGO stage I/II compared to 18.4% (36/196) of patients in FIGO stage III/IV, and the difference is significant (*p* = 0.019). For histological grade, 0% (0/19) of well differentiated EOC samples was CD117 positive in fibroblast-like stromal cells, compared to 11.3% (7/62) in moderately differentiated EOC samples and 21.1% (28/133) in poorly differentiated EOC samples. Thus, CD117 immunoreactivity was closely associated with differentiation grade (*p* = 0.010). Ovarian serous carcinomas and the group of undifferentiated EOC, mixed EOC and others showed more CD117-positive fibroblast-like stromal cells than the group of mucinous EOC, endometrial EOC and clear cell carcinoma (*p* = 0.012).

**Table 2:** Associations between CD117 expression in fibroblast-like stromal cells and clinicopathological features (N = 242).

		CD117 expression, n (%)		
	Total N	negative	positive	*p* value
Age group				0.411
≤59	121	104 (86.0)	17 (14.0)	
≥60	111	91 (82.0)	20 (18.0)	
missing	10			
FIGO stage				0.019
I+II	46	44 (95.7)	2 (4.3)	
III+IV	196	160 (81.6)	36 (18.4)	
Differentiation grade				0.010
well	19	19 (100)	0 (0)	
moderately	62	55 (88.7)	7 (11.3)	
poorly	133	105 (78.9)	28 (21.1)	
missing	28			
Histological subtype				0.012
serous	163	131 (80.4)	32 (19.6)	
MUC+END+CLE	48	47 (97.9)	1 (2.1)	
UND+MIX+others	27	23 (85.2)	4 (14.8)	
missing	4			

MUC, Mucinous carcinoma; END, Endometrioid carcinoma; CLE, Clear cell carcinoma; UND, Undifferentiated carcinoma; MIX, Mixed carcinoma.

### CD117 expression in EOC cells and clinical impact

For CD117 expression in EOC cells, we did not observe any statistical difference in different age groups (*p* = 0.632), FIGO stage groups (*p* = 0.267), differentiation grade groups (*p* = 0.306) or histological subtype groups (*p* = 0.439) (Table 3).

**Table 3:** Associations between CD117 expression in EOC cells and clinicopathological features (N = 242).

		CD117 expression, n (%)		
	Total N	negative	positive	*p* value
Age group				0.632
≤59	121	109 (90.1)	12 (9.9)	
≥60	111	102 (91.9)	9 (8.1)	
missing	10			
FIGO stage				0.267
I+II	46	44 (95.7)	2 (4.3)	
III+IV	196	176 (89.8)	20 (10.2)	
Differentiation grade				0.306
well	19	17 (89.5)	2 (10.5)	
moderately	62	59 (95.2)	3 (4.8)	
poorly	133	116 (87.2)	22 (12.8)	
missing	28			
Histological subtype				0.439
serous	163	149 (91.4)	14 (8.6)	
MUC+END+CLE	48	42 (87.5)	6 (12.5)	
UND+MIX+others	27	26 (96.3)	1 (3.7)	
missing	4			

MUC, Mucinous tumor; END, Endometrioid carcinoma; CLE, Clear cell carcinoma; UND, Undifferenciated tumor; MIX, Mixed epithelial tumor.

### Survival analyses

**Figure 5:**
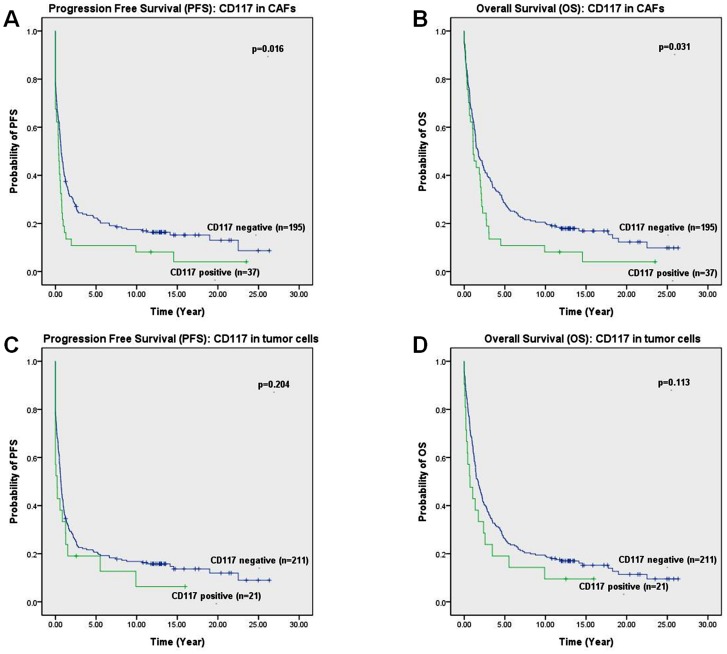
The survival probabilities of CD117 expression in ovarian carcinoma cells and fibroblast-like stromal cells were demonstrated. PFS (A) and OS (B) probabilities are shown for the group of CD117 positive and negative in fibroblast-like stromal cells. CD117 negativity in stromal cells shows a significantly better survival probabilities (*p*<0.05). PFS (C) and OS (D) probabilities are revealed for CD117 expression in ovarian carcinoma cells. No significant difference was observed for survival probabilities between CD117+ tumor cell group and CD117- tumor cell group (*p*>0.05).

Progression free survival (PFS) and overall survival (OS) were used to analyze survival time in our study (Table 4). The patient group with CD117 expression in fibroblast-like stromal cells had a significantly shorter OS (Figure 5A) and PFS (Figure 5B) than the patient group not expressing CD117 in fibroblast-like stromal cells.

**Table 4:** CD117 expression and survival (years).

	Mean		Median		*p* value
	Extimate	95% CI	Extimate	95% CI	
OS:CD117 in tumor cells					
Negative (n = 211)	5.649	4.494 to 6.805	1.719	1.267 to 2.172	0.113
Positive (n = 21)	2.995	0.948 to 5.041	0.715	0.000 to 1.652	
OS:CD117 in fibroblast-like stromal cells					
Negative (n = 195)	5.922	4.686 to 7.159	1.719	1.165 to 2.274	0.031
Positive (n = 37)	2.978	1.269 to 4.688	1.098	0.605 to 1.591	
PFS:CD117 in tumor cells					
Negative (n = 211)	4.592	3.405 to 5.774	0.665	0.516 to 0.814	0.204
Positive (n = 21)	2.274	0.332 to 4.217	0.216	0.000 to 0.560	
PFS:CD117 in fibroblast-like stromal cells					
Negative (n = 195)	4.484	3.574 to 6.105	0.706	0.483 to 0.929	0.016
Positive (n = 37)	2.180	0.415 to 3.946	0.397	0.221 to 0.573	

When comparing the two patient groups with positive and negative CD117 expression in tumor cells, we did find a trend for the CD117-positive group to have a worse OS (Figure 5C) and PFS (Figure 5D) probability, but no statistical significance was achieved.

## Discussion

In our study 9% of EOC cases expressed CD117 in carcinoma cells, with a relatively lower positive frequency compared to 15% positivity in ovarian serous carcinoma in a previous study [15]. Garrity and coworkers [16] have pointed out the variable positivity using different antibodies, showing 6% positivity using rabbit anti-human CD117 antibody from one company compared to 33% positivity with stronger staining background using rabbit anti-human CD117 from another antibody. In our current study, we firstly tested 4 commercial anti-human CD117 antibodies and chose the optimized one with high specificity and low background staining as revealed by the previous study [17]. Further IHC procedure optimization was performed in order to ensure high sensitivity for this antibody as well.

CD117 is mostly reported in mast cells/myeloid cells (mastocytoma/acute myeloid leukemia) [18], germ cells (seminoma) [19], Cajal cell (gastrointestinal stromal tumors) [20], [21] and some epithelial cells [22], [23], [24], [25]. CD117 expression in tumor stromal cells is neglected and rarely reported. In our series, we disclosed for the first time that CD117 expression in fibroblast-like stromal cells had a statistical association with poor clinical outcomes.

The importance of microenvironment for tumor growth is well established [26], [27], [28], [29]. Fibroblast cells, as predominant component in the microenvironment, act synergistically with carcinoma cells to prepare the microenvironment according to the “seed and soil” theory [29], [30]. Fibroblasts in mammals comprise a highly heterogeneous group of cells, and reflect a substantial genetic diversity [31]. Similar to organ fibrosis, the fibroblasts at the site of a carcinoma remain perpetually activated, and this subpopulation of fibroblasts are designated cancer-associated fibroblasts (CAFs) [32]. FAP and α-SMA are widely used among other markers to mark CAFs in tumors. FAP is selectively expressed on fibroblasts within the tumor stroma or on CAFs [33], [34]. α-SMA is positive in most of smooth muscle cells and fibroblasts in the stroma of the epithelial cancer [35], [36]. In our study, the CD117+ fibroblast-like stromal cells were neither positive for FAP nor positive for α-SMA, which shadows forth that these morphologically fibroblast-like stromal cells were not supposed to be fibroblasts actually.

To further identify these cells, mesenchymal stem/stromal cell (MSC) marker CD73 (NT5E) were immune-evaluated. More intriguingly, All CD117+ fibroblast-like stromal cells were positive for CD73. Mesenchymal stem cells (MSCs) were initially isolated from the bone marrow and demonstrated the multipotency to differentiate into a variety of cell types [37], [38]. MSCs recruited into the tumors as the progenitors of stromal cells may play a significant role in the regulation of both solid and haematological malignancies [39]. It is still an open discussion about how to define MSCs, because no single marker is specific to identify MSCs to date [40]. Cultured MSCs are uniformly and strongly positive for CD105, CD90, and CD73, regardless of their passage or time in culture [41], and it has been one of the minimal criteria to identify MSC [40], [42]. Moreover, the morphology of MSCs was reported to be fibroblast-like [43], [44]. Bone marrow-derived MSCs selectively express FAP but not other resources [45], and slightly express α-SMA [46]. But MSCs in tumor stroma are currently not reported to express these two markers. As a result, to our point of view, there is a possibility for these CD117+/CD73+ fibroblast-like stromal cells to be MSC-derived, although currently we are not able to confirm this suppose.

The effect of MSCs on tumor growth is still controversial, but Ljujic, et. al believes MSCs are capable to home to tumor cites and promote tumor growth in mice [47]. CD73, also known as ecto-5′-nucleotidase, is an enzyme that in humans is encoded by the NT5E gene and commonly serves to convert adenosine monophosphate (AMP) to adenosine. Through the increasing adenosine, CD73 contribute to immunosuppressive effects of anti-tumor T cells [48] and regulate adaptive responses upon hypoxia[49]. Furthermore, CD73 expression in tumor cells and tumor environment are required for tumor angiogenesis [50].The percentage of CD73+ Natural killer (NK) NK cells increases significantly on coculture with MSCs and thus acquires the ability to convert AMP into adenosine [51]. Epithelial mesenchymal transition (EMT) is a fundamental biologic process during which epithelial cells lose their polarity and change to a mesenchymal phenotype [52], [53], [54]. The mesenchymal state facilitates cells with the capacity of migrating to distant organs and maintain stemness, allowing the initiation of metastasis [53]. CD73 may contribute to induce EMT by the regulation of EMT-related key factors including cadherin-1 (CDH1) and vimentin (VIM) [55].

The traditional defined MSCs, including bone marrow-derived MSCs (BM-MSCs) and human embryonic stem cell-derived mesenchymal stroma cells (hES-MSCs), express CD73 but rarely express CD117 [56], [57]. On the other hand, CD117 was also regarded as one of MSC markers [11], [58]. In our study, we found that CD117 expressed in fibroblast-like stromal cells in ovarian carcinomas was significantly linked to poor clinical characters and survival time. Although these CD117 positive stromal cells were negative for both fibroblast markers FAP and α-SMA, it was confirmed in our study that these cells were positive for mesenchymal stem cell marker CD73, which strongly indicates their mesenchymal stem cells in nature. To the best of our knowledge, this is the first report of CD117+/CD73+ tumor stromal cells with negative clinical consequences.

To conclude, it is verified in our study that CD117+/CD73+ fibroblast-like stromal cells are significantly associated with poor clinical manifestations and poor survival probability in ovarian carcinomas, but CD117 expression in tumor cells does not show any clinical significance. Thus, it is worthy of further study for CD117 positive and CD73 positive stromal cells in EOCs in order to explore their potential application in prognostic prediction and targeting therapy.
