# Expression of concern: CD117 expression in fibroblasts-like stromal cells indicates unfavorable clinical outcomes in ovarian carcinoma patients

**DOI:** 10.1371/journal.pone.0339147

**Published:** 2025-12-18

**Authors:** The PLOS One Editors

After this article [1] was published, concerns were raised regarding results presented in Fig 4. Specifically, the upper left region of panel A appears to overlap with the lower right region of panel C. The first author RH acknowledged this similarity but stated that the original images are no longer available, and therefore were unable to verify whether Figs 4A and 4C originated from the same sample. They stated that the slides shown in these panels are intended to be representative of CD117 or CD73 stained cells.

Contrary to the declaration in the Data Availability statement, the original raw data files supporting the article’s results were not provided with the article, and first author RH stated these data are no longer available. As the original raw data files supporting the article’s results are no longer available, this article [1] does not comply with the PLOS Data Availability policy, and therefore, the *PLOS One* Editors issue this Expression of Concern.
